# PeakForce AFM Analysis Enhanced with Model Reduction Techniques

**DOI:** 10.3390/s23104730

**Published:** 2023-05-13

**Authors:** Xuyang Chang, Simon Hallais, Kostas Danas, Stéphane Roux

**Affiliations:** 1 Université Paris-Saclay/CentraleSupélec/ENS Paris-Saclay/C.N.R.S., LMPS—Laboratoire de Mécanique Paris-Saclay, 91190 Gif-sur-Yvette, France; xuyang.chang@ens-paris-saclay.fr; 2 LMS, C.N.R.S., École Polytechnique, Institut Polytechnique de Paris, 91128 Palaiseau, France; simon.hallais@polytechnique.edu (S.H.); konstantinos.danas@polytechnique.edu (K.D.)

**Keywords:** PeakForce-QNM, AFM, POD, pattern recognition, clustering analysis, manifold learning

## Abstract

PeakForce quantitative nanomechanical AFM mode (PF-QNM) is a popular AFM technique designed to measure multiple mechanical features (e.g., adhesion, apparent modulus, etc.) simultaneously at the exact same spatial coordinates with a robust scanning frequency. This paper proposes compressing the initial high-dimensional dataset obtained from the PeakForce AFM mode into a subset of much lower dimensionality by a sequence of proper orthogonal decomposition (POD) reduction and subsequent machine learning on the low-dimensionality data. A substantial reduction in user dependency and subjectivity of the extracted results is obtained. The underlying parameters, or “state variables”, governing the mechanical response can be easily extracted from the latter using various machine learning techniques. Two samples are investigated to illustrate the proposed procedure (i) a polystyrene film with low-density polyethylene nano-pods and (ii) a PDMS film with carbon–iron particles. The heterogeneity of material, as well as the sharp variation in topography, make the segmentation challenging. Nonetheless, the underlying parameters describing the mechanical response naturally offer a compact representation allowing for a more straightforward interpretation of the high-dimensional force–indentation data in terms of the nature (and proportion) of phases, interfaces, or topography. Finally, those techniques come with a low processing time cost and do not require a prior mechanical model.

## 1. Introduction

Atomic force microscopy (AFM) has been primarily used as an imaging tool (e.g., contact mode) to determine the morphological features, such as surface topography, at a very high spatial resolution [1,2]. Later, AFM functionality was extended to scanning probe microscopy (SPM) [3] through different modes: e.g., tapping mode [4], pulse force mode [5] and contact resonance mode [6]. These extensions [7] allowed for measuring force–indentation curves related to the local tip–sample interaction, giving access to various mechanical properties (e.g., adhesion, dissipation, stiffness, etc.) naturally registered with topography at the exact same point. Though much information is gathered, these classical AFM modes are typically slow, which makes any detailed scan impractical.

A decade ago, the PeakForce quantitative nanomechanical (PF-QNM) technique [8] was introduced and designed as a new extension of the pulse force mode to increase the scanning speed, leading to a significant increase in data throughput. In this particular AFM mode, the tip is intermittently brought into contact with the sample by monitoring the cantilever deflection, thus avoiding any potential surface damage and greatly reducing the lateral force during tip–sample interaction. More importantly, the force–indentation data are generated separately for each tip oscillation (pixel per pixel) inside the region of interest (ROI), whereby the applied maximum force of the nano-indentation (the so-called PeakForce) can be held constant, allowing for quantitative, robust and detailed mapping of various mechanical properties as well as on-the-fly topography comparison [9,10,11,12]. This AFM mode is a major step forward in characterizing heterogeneous multi-phase materials at the nanometre scale.

It is important to emphasize that in the PeakForce-QNM AFM mode, the tip–sample interaction is a combined effect of the effective contact regime, the material physical and chemical properties and the sample topography [13,14,15]. Most data analysis algorithms for AFM post-processing have been developed to achieve a reliable estimate of the measured modulus [16,17], and their applications are mostly limited to intrinsically stiff and non-viscous materials (e.g., metals and ceramics). However, due to the non-uniform loading rate of the AFM tip during PF-QNM scanning, the analysis of viscoelastic materials, such as polymer and polymer-based composites (e.g., PMMA, PDMS), becomes extremely challenging for most modulus-based data analysis and segmentation methods [18].

Moreover, PeakForce-QNM AFM datasets often consist of many pixels (up to 512×512), each containing many features (the corresponding force at any given level of indentation). Though accessing such an abundance of information for all pixels is highly beneficial to generalize the AFM mapping, handling so much data—some of which may be irrelevant or even misleading (due to either viscoelasticity or topography)—is a typical burden to the algorithm of built-in PF-QNM segmentation and classification. Therefore, the computational cost of analysis at the tip–surface force–indentation data calls for a robust and efficient method in finding out which features are the most relevant to understand the heterogeneous structure of the investigated sample without any prior knowledge or assumptions.

Machine learning clustering analysis techniques (such as K-means analysis), in AFM post-analysis, have already been pioneered by [19,20]. However, the use of machine learning algorithms is devoted to particular properties for clustering analysis (e.g., topography and phase lag in the work of [19], topography, PeakForce and adhesion in [20]).

Alternatively, one can consider that the entire mechanical force–indentation responses of different pixels, achieved by AFM mapping, are actually sampled from a low-dimensional manifold (i.e., nature of phase, local topographical features, effective contact area, etc.) that is embedded in a high-dimensional space (the entire force–indentation response). In the following the mechanical response is sampled over $N_{\delta }=512$ data points. This discretized response can be seen as a vector in this $N_{\delta }$-dimensional space. Although each pixel consists of many ($N_{\delta }$) features, it may be simply described as a function of only a few underlying parameters. However, the high dimensionality of the initial dataset (the force–indentation curves) poses great challenges to standard manifold learning. In the presence of many irrelevant features, learning models are prone to overfitting, thus becoming less powerful [21].

In the previous work [22], proper orthogonal decomposition (POD [23], also known as SVD [24] or PCA [25]) was shown to be an efficient way to ‘visually’ uncover these “state variables” by offering a low-dimensional representation of the entire force-mechanical data. By selecting the most relevant POD modes as the subset to represent the initial AFM data, in the subspace generated by POD (usually 2D or 3D representations), the user can have an intuitive visual inspection of the corresponding data manifold, which is of great practical interest. In this work, as a step forward, we attempt to go beyond visualization, through a so-called “machine learning process”, to directly identify the underlying “state variables” governing the AFM force–indentation dataset.

In general, the clustering analysis assumes that there exists a discrete set of behaviours that can be labelled from their centroids, whereby the latter needs to be interpreted. In the present work, we will deal with two popular approaches:

- clustering analysis;
- manifold reparametrization.

In this context, many variants exist, either those based on a similarity classification in a Euclidean-metric space (e.g., K-means [26], fuzzy c-means [27,28]), or those using probabilistic modelling (e.g., Gaussian mixture model [29]) which assume the presence of different datasets. In the latter, each Gaussian component is characterized with a probability distribution function which needs to be determined, say, from their maximum likelihood. For manifold reparametrization, the subset in the POD subspace may form a curve (one-dimensional manifold) or a surface (two-dimensional manifold), or just several discrete points (zero-dimensional manifolds), which become equivalent to the clustering analysis. For an n-dimensional manifold, one may propose an n-coordinate parameterization (the prototype of which is isometric feature mapping, also known as an isomap [30]) to smoothly interpolate between different responses. Then, extreme points may be selected along this manifold to allow for model-based interpretation, a much easier (and lighter) task compared to raw QNM data analysis.

The present work presents the use of the above-mentioned “classical” algorithms, as available in different toolboxes, (Matlab was used in this study), to the novel context of voluminous data achieved from PF-QNM AFM measurements. After appropriate editing and formatting, the raw initial data is firstly compressed into a subset with a much lower dimensionality through proper orthogonal decomposition [22]. The subset is then visually inspected inside the POD subspace. Different machine learning tools from those listed previously may be adopted depending on the dimensionality of the observed data manifold.

The novelty and benefits of using the combined model reduction and machine learning algorithm are summarized as follows; Firstly, the entire force–indentation data are used instead of a particular material property dictated by one single point of the curve (e.g., adhesion, topography, etc.). Moreover, the interpretation of machine learning results, in terms of mechanical properties, nature of phases, and topography, is now limited to the identified manifold (or characteristic points over this manifold) to avoid any potential bias. Finally, the proposed approach is agnostic to the physics of the surface–tip interaction. It makes the method very powerful and versatile as no prior knowledge or assumptions are required, even though it eventually calls for a final interpretation of the data and results of the clustering analysis. Nevertheless, the clarity of the obtained patterns allows us to give a robust mechanical interpretation of the classification results. In particular, the proposed algorithm permits us to easily visualize and identify the underlying parameters of the AFM data (e.g., nature of phases, sharp variation in topography, interfaces, etc.), within a short processing time and with no prior mechanical model.

The rest of the paper is organized as follows. Section 2 recalls the principles of PeakForce AFM measurements. Section 3 introduces the combined proper orthogonal decomposition (POD) and machine learning (POD-ML) algorithm in order to analyse voluminous AFM data. Section 4 shows the performance of the proposed algorithm over two different heterogeneous samples: the first consists of a polystyrene (PS) matrix onto which low-density polyethylene (LDPE) disks have been deposited; the second sample is made of hard carbon–iron particles randomly embedded into a polydimethylsiloxane (PDMS) matrix. This second composite material is also known as a magnetorheological elastomer (MRE) since the embedded particles have the ability to significantly rearrange themselves upon the application of an external magnetic field [31] or change their magnetic properties if subjected to large strains [32]. Section 5 draws the main conclusions.

## 2. Theoretical Background of the PeakForce-QNM Mode

A brief presentation of PeakForce AFM is first presented. Interested readers can refer to [10,22] for more details.

The specimen is placed over a support with holes, under which a low vacuum holds the sample without mechanical grips. The entire AFM is placed inside a box where the temperature is held constant with great care. The system is initially thermalized for a long time before starting the measurements. Thus, conditions are carefully set to limit drift issues, and no sign of this could be seen in the collected data.

At each pixel, an elementary “mechanical test” is performed by moving the tip, contacting the surface, pressing to a desired force, and withdrawing the tip to free it from the surface. This mechanical probing test lasts about 0.5 ms. Thus, even if a slow drift takes place, this would not be seen over such a short time. This would manifest itself in a biased positioning of the pixel onto the surface, with negligible consequences on the proposed analysis.

**Figure 1:**
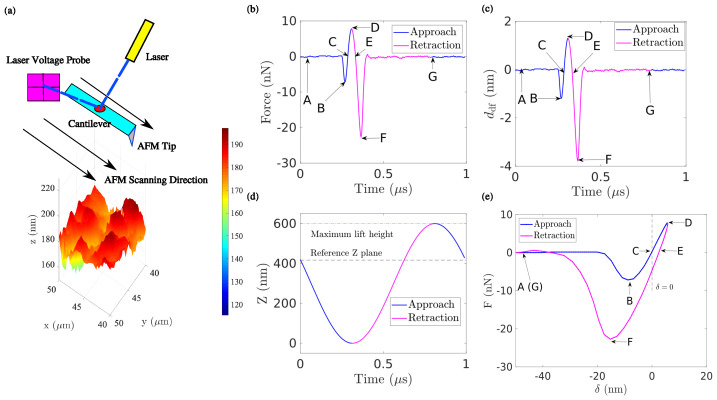
Scheme of PeakForce-QNM mode. The surface is raster scanned, and at each point, an elementary mechanical test is performed. (**a**) As the AFM head is moved vertically, the tip position is measured from the reflection of a laser, and the tip force F is evaluated from the deflection of cantilever. Thus, the force F(t) (**b**) and deflection $d_{\mathrm{df}}\left(t\right)$ (**c**) are measured over time. From the monitored AFM cantilever position Z(t) (**d**), the indentation depth $\delta =Z-d_{\mathrm{df}}$ can be evaluated, and the mechanical response of the contact, F(δ), is recorded as shown in (**e**). The different letter labels are characteristic points mentioned in the main text.

The operation of the PeakForce AFM mode is compactly illustrated in Figure 1. At ambient room temperature, the laser spot is focused on the surface of the AFM cantilever (Figure 1a) and the associated photo-diode probe measures the corresponding laser shifting voltage (LSV) over time, δV(x,t), at a given position on the surface described by the in-plane position vector x=(x,y). After a proper calibration process of the AFM cantilever (usually performed on a rigid (non-deformable) sapphire sample), its bending stiffness κ and the sensitivity of the cantilever deflection γ can be properly estimated, allowing the user to convert the measured laser shifting voltage (LSV) signal into the instantaneous AFM cantilever reaction force F(x,t) (Figure 1b) and the associated cantilever deflection $d_{\mathrm{df}}\left(x,t\right)$ (Figure 1c) at each sampling time step t via the relations

(1) $$\begin{array}{c} F(x,t)=\kappa \delta V(x,t) \end{array}$$

(2) $$\begin{array}{c} d_{\mathrm{df}}\left(x,t\right)=\gamma \delta V\left(x,t\right) \end{array}$$

In turn, the actual indentation depth δ(x,t) of the cantilever tip may be computed as the difference between the prescribed vertical displacement of the cantilever Z (Figure 1d) and the cantilever deflection as $d_{\mathrm{df}}$, i.e.,

(3) $$\begin{array}{c} \delta \left(x,t\right)=Z-d_{\mathrm{df}}\left(x,t\right). \end{array}$$

Figure 1e shows a representative force–indentation response, F−δ, at a fixed position (pixel) x. The use of F−δ, instead of force–displacement (F−D curve) or of time *t*, allows to eliminate almost entirely the influence of topography during the AFM nano-indentation. The paths A→B→C→D (blue line) and D→E→F→G (red line) correspond to the loading (approach) and unloading (retract) response, respectively.

The entire F−δ response may be further divided into four main regimes governed by different mechanisms (Figure 1e):

—Regime I, A→B, ‘snap-in’: As the tip approaches the surface of the specimen, an unstable jump towards contact occurs.

—Regime II, B→C→D, ‘adhesive contact (indentation)’: The first force minimum during loading at B is initially considered a conventional definition of contact [33], used in the Bruker definition of topography. Later, it is commonly accepted to consider point C as the reference for topography to avoid the intrinsically unstable character of this “snap-in” and its associated hysteresis [34].

At point C, the tip is in contact with the surface and exerts an overall zero applied force, obeying the following implicit equation:

(4) $$F\;(x,\delta_{e}\left(x\right)=0)=0\;.$$

As the tip is pushed towards the surface, the reaction force turns from attractive (before point C) to repulsive (after point C) until reaching a maximum force at point D.

—Regime III, D→F, ‘adhesive contact (retraction)’: The tip is then withdrawn from the specimen (unloading), and the response is that of (visco)-elastic adhesive contact. Adhesion can be characterized through the maximum pull-out force F reached at point F in the F−δ curve (dark blue star symbol).

—Regime IV, F→G, ‘snap-off’: The complete retraction of the tip is mainly dominated by the mechanical instability of tip detachment from the surface, similar to Regime I, but with a higher amplitude because of adhesion.

Before proceeding further, it is useful to present a general discussion of the most frequently used mechanical/physical features that can be measured during AFM nano-indentation.

—‘Elastic modulus’ When the maximum penetration depth is controlled to be constant during the scan, the maximum (compressive) load $F_{\max}$ is a ‘qualitative’ indicator of stiffness. However, the latter quantity can hardly be trusted due to numerous sources of mechanical non-linearity, surface roughness, and the inherent difficulty in achieving perfect control during high-rate scans. A much more reliable stiffness estimate can be obtained by considering the unloading from D→F. Using a spherical or conical elastic contact model, an effective apparent Young’s modulus can be estimated by fitting the force–indentation curve. In particular, the initial slope of unloading dF/dδ is a good indicator of the stiffness, which can be used to evaluate the elastic properties if the contact area is known, and this is where a model exploiting tip shape is needed. However, this analysis becomes more difficult when the probed material displays a viscous or visco-elastic behaviour during AFM indentation (e.g., polymers often exhibit significant strain rate-dependent properties). Therefore, most analyses of the elastic modulus are strongly user-dependent and can lead to erroneous results, as has been recently shown in [22] for carbonyl–iron particles embedded in a PDMS matrix (see also Section 4.2 in the present work).

—‘Adhesion (maximum pull-out force)’ Based on the DMT (Derjaguin–Muller–Toporov) contact model [35], the adhesion is directly related to the maximum pull-out force, $F_{\mathrm{adh}}$ (point E). It reflects the inter-facial energy between two contact bodies (governed by van der Waals, electrostatic, or capillary forces) δγ. They are related through the effective contact radius $a_{\max}$,

(5) $$F_{\mathrm{adh}}=-2\pi a_{\max}\delta \gamma$$

The original DMT solution only considered the spherical contact between the tip and the planar specimen, but the same technique can be equally applied to any complex geometry for which the corresponding boundary conditions are provided. In a qualitative sense, the presence of a sharp topography gradient usually leads to a local decrease in adhesion.

—‘Dissipation (viscosity)’: The dissipation measures the mechanical energy dissipated between the indentation (C→D) and retraction force–indentation curves (D→E). In the absence of other irreversible phenomena, the dissipated energy from C to E can provide an estimate for the viscosity of the material,

(6) $$E\left(x\right)=\int_{0}^{\delta_{\max}\left(x\right)}\left(F_{\mathrm{indent}}\left(x,\delta \left(x\right)\right)-F_{\mathrm{retract}}\left(x,\delta \left(x\right)\right)\right)d\delta \left(x\right)$$

In a simpler fashion, the difference between the two points C and E, i.e., the position $\delta_{0}$ at which $F(\delta_{0})=0$ during indentation and retraction, is a good indicator of such viscous effects. Alternatively, the (attractive) force at zero indentation depth during retraction, F(δ=0), provides similar information. In the case of a purely elastic response (even including non-linearities), E=0, $\delta_{0}=0$, and F(δ=0)=0. Let us, however, stress that any meaningful relative comparison of these indicators at different probed points depends on a well-controlled maximum penetration depth (or maximum compression force).

## 3. Combined Proper Orthogonal Decomposition and Machine Learning Algorithm

### 3.1. An Introductory Example about Pattern Recognition

The segmentation of the dataset F(x,δ) is a typical one in pattern recognition problems. In a two-phase heterogeneous medium, there are two general classes (e.g., the nature of phases) to be distinguished, say “black” and “white”. Based on the nature of the selected materials, the contrast between different phases can be seen as the difference in stiffness, adhesion, or both of them (the definition of “black” or “white”). Moreover, the class of each object may be subdivided into sub-species based on other higher-order parameters. For instance, a specific class (phase) can be further divided into two different topography states: smooth or rough. In this regard, pattern recognition may be characterized as an information labelling process (“a data classifier”).

In summary, when assessing AFM data, the adequate pattern recognition process should be able to achieve the following objectives:

- to segment any mechanically relevant phases;
- to identify the interface between each phase;
- to refine the segmentation if required, highlighting the contribution of higher-order parameters, such as topography variations.

### 3.2. The General Framework of the POD-ML Combined Algorithm

**Figure 2:**
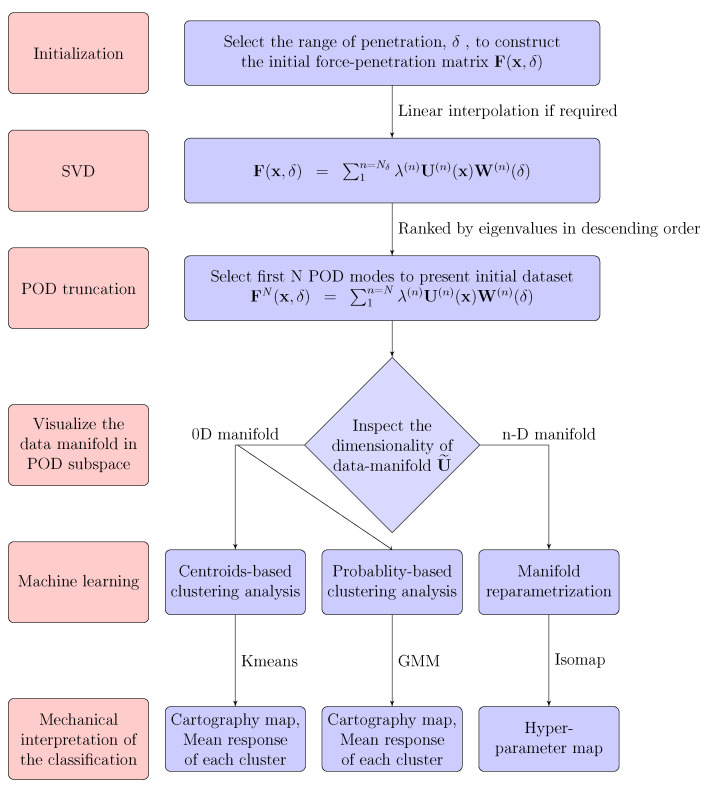
General framework of the combined POD and ML algorithm.

In order to identify interesting patterns from voluminous AFM data, a combined POD and ML algorithm is introduced in the following (Figure 2).

—Data clipping and re-sampling: When assessing the heterogeneous multi-phase specimen using PF-QNM AFM modes, conventionally, the maximum load is set as constant for every pixel x inside the region of interest (ROI); hence, a different penetration depth is expected for heterogeneous samples. Moreover, this control may become difficult for fast scans, especially at interfaces where a strong stiffness contrast is expected. Thus, exploiting the entire force–penetration response is extremely subtle and prone to spatial inconsistencies.

To guarantee a meaningful physical comparison of force–indentation curves among different pixels, it is proposed, at a post-processing stage, to investigate the force–indentation response, F(x,δ), only inside a given range of δ (common to all pixels), where the experimental data is “clipped”. Following the above discussion on the mechanical response, we chose to focus on the retraction phase and consider a range of δ values with positive penetration (to easily identify viscous dissipation from the displacement at F=0), which also includes the peak attractive force for most pixels to capture adhesion too. For the following analysis, the force–indentation response, F(x,δ), is sampled along a predetermined set of δ values, and a linear interpolation is used to evaluate the corresponding values of F from the effectively sampled values. The fine sampling of the response ($N_{\delta }=512$) is such that the interpolation scheme makes no difference in the following. The data are thus discretized to a matrix format, convenient for the subsequent processing.

—Information reduction: Next, the model reduction techniques (e.g., POD) are used to achieve a low-dimensional representation from the initial dataset, where the data manifold can be visually inspected, allowing the user to “observe” the underlying state variables.

The AFM data are considered as a force–indentation matrix, F(x,δ), with size $N_{x}\times N_{\delta }$, where $N_{x}$ is the total number of pixels inside the ROI and $N_{\delta }=512$ stands for the total number of sampled δ values for each pixel. Note that in practice, the number of pixels is much larger than that of sampling δ values, $N_{x}\gg N_{\delta }$. The force matrix is thus decomposed into $N_{\delta }$ POD modes as shown in Equation (7). Each POD mode is written as a multiplication of its eigenvalue $\lambda^{\left(n\right)}$, the associated eigen–force–indentationresponse $W^{\left(n\right)}\left(\delta \right)$ and corresponding spatial modulation $U^{\left(n\right)}\left(x\right)$, such that

(7) $$F\left(x,\delta \right)=\sum_{n=1}^{N_{\delta }}\lambda^{\left(n\right)}U^{\left(n\right)}\left(x\right)W^{\left(n\right)}\left(\delta \right).$$

Guided by the eigenvalue amplitude, one can seamlessly reconstruct the force–indentation response by selecting the first N POD modes starting from the larger eigenvalue while discarding higher-order modes associated with smaller eigenvalues since the latter contribute with negligible power to the response (in the sense of an L2 norm). This notion allows for approximating the original force–indentation data by truncating the first N POD eigenmodes as

(8) $$F\left(x,\delta \right)\approx F^{N}\left(x,\delta \right)\equiv \sum_{n=1}^{n=N}\lambda^{\left(n\right)}U^{\left(n\right)}\left(x\right)W^{\left(n\right)}\left(\delta \right).$$

The selected subset ${\tilde{U}}^{n}=\left[U^{\left(1\right)},U^{\left(2\right)},\cdots ,U^{\left(n\right)}\right]$ can provide an initial visual inspection of the data manifold in the subspace generated by the POD with a much lower dimensionality $R^{N}$ (usually a N= two or three-dimensional representation, and certainly $N\ll N_{\delta }$).

—Information mapping: The reduced data manifold generated by the POD may form a curve (one-dimensional manifold), a surface (two-dimensional manifold) or just several discrete points (zero-dimensional manifold). To identify the underlying state variables, depending on the dimensionality of the manifold, different segmentation tools can be selected:

- zero-dimensional manifolds: The data are grouped into several discrete clusters in the POD subspace. Clustering analysis is recommended in this case. K-means is the simplest choice, but requires good initialization. A probability-based clustering analysis, such as provided by the Gaussian mixture model (GMM), is an interesting alternative that gives additional information: not only centroids, but also statistical (co-)variance of each cluster.
- n-dimensional manifold: One may propose an n-coordinate manifold re-parameterization to smoothly interpolate between different responses.

However, sometimes the choice between these two cases is ambiguous. Such is the case for the second example (CIP/PDMS) discussed in Section 4.2 where hard particles are present in a soft matrix. Even if two well-populated groups can be identified as these two components, a continuum of responses may be seen. This could correspond physically to a thin layer of polymer coating the hard particles. Depending on the layer thickness, a mechanical response intermediate between the particles and matrix would be observed. For this case, the two options will be documented. The first one (Section 4.2.1) corresponds to the zero-dimensional and a GMM approach will be shown to lead to a meaningful segmentation but also to the overlap of different Gaussian clusters suggesting an artificial partition in distinct groups. In turn, the second option of the n-dimensional strategy (Section 4.2.2) will be shown to be more appropriate. Manifold re-parameterization will then be discussed for the very same case. This will illustrate that the ambiguity between these two approaches, that may be physically justified, does not prevent the machine learning tools from providing each a fair representation, faithful to reality, yet requiring the user to provide a proper physical/mechanical interpretation.

—Information labelling: An interpretation (e.g., mechanical, chemical, etc.) of the segmentation results is required. It also helps the user to validate the result of the classifications.

The previous general methodology is applied and further discussed using examples in the following sections. The first case (PS-LDPE sample) is a test case for the approach since it deals with a well-separated two-phase nano-surface. The second example is used to reveal the efficiency of the proposed approach in a substantially more complex composite material, that of PDMS comprising carbonyl–iron particles (CIP). The segmentation algorithms are presented in detail in the first example and directly applied in the second.

## 4. Results and Discussion

### 4.1. Sample of PS-LDPE

#### 4.1.1. Description of the Sample

A sample made from low-density polyethylene (LPDE) well-separated nanopods deposited on a polystyrene (PS) thin substrate is investigated. More details about the two investigated samples can be found in [22]. This sample is a benchmark in our work because of its simplicity and universal usage to calibrate AFM tips (Bruker RTESPA-150 type). For the AFM mapping, an ROI consisting of $5\times 5\;\mu m^{2}$ is scanned with a spatial resolution of 64×64 points and a standard acquisition frequency of 1 kHz.

#### 4.1.2. POD Truncation

**Figure 3:**
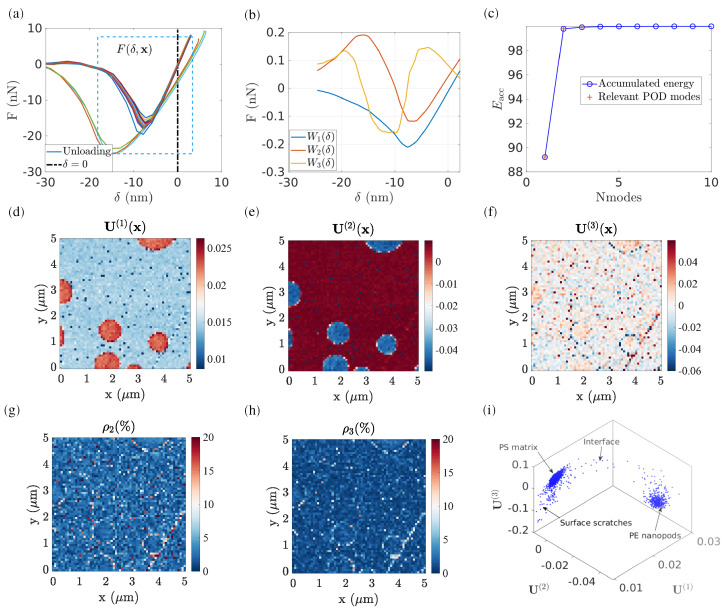
(**a**) Arbitrarily selected force–indentation response at various pixels during retraction; the rectangle indicates the region selected for POD analysis. (**b**) First three POD elementary force modes as a function of penetrations. (**c**) Accumulated “energy” (L2-norm of reconstructed signal compared with the raw data) as a function of the number of POD modes, the first three POD modes represent 99% of the energy. (**d**) First POD spatial mode revealing the phases. (**e**,**f**) Second and third POD modes revealing more subtle information such as PS–LPDE interfaces and showing higher-order features related to surface roughness. (**g**,**h**) Residual error resulting by only keeping the first N modes to describe the force–indentation response at each pixel, N=2 and 3, respectively. (**i**) Subspace generated by the first three POD modes $\left[U^{\left(1\right)};U^{\left(2\right)};U^{\left(3\right)}\right]$.

Figure 3a shows the representative force–indentation response by selecting twenty random pixels inside the ROI. It is clear that all pixels can be classified into two main data clusters: the first exhibits a stiffer response with low adhesion and negligible viscosity; whereas the second shows a softer response with a much stronger adhesion and more pronounced viscosity (as indicated by the hysteresis between loading and unloading).

Subsequently, the cropped force–indentation data points (ranging from δ=−15 nm to 5 nm) are selected and decomposed into 3 POD modes. Let us note that for some “pixels”, contact seems not to be set or detected, and these points are disregarded from the following analysis. Figure 3b plots the first three POD elementary force modes $W^{\left(n\right)}$ as a function of penetration. Figure 3c illustrates the power contribution of the first N POD modes. These first three POD modes represent 99% of the original measured F−δ response in terms of “power”, leading respectively to the values, $\tau_{1}=89\%$, $\tau_{2}=8\%$, and $\tau_{3}=0.4\%$. Figure 3d–f show the first three POD spatial modes $U^{\left(n\right)}\left(x\right),n\leq 3$, ranked by their associated eigenvalue $\tau_{n}$ in descending order.

The POD analysis is extremely efficient in hierarchically reorganizing the data:

*τ_n_*: the associated eigenvalue represents the power (in the sense of an L2 norm) of the n-th POD mode in the total matrix. The accumulated power $E_{\mathrm{acc}}$ represents the total power contribution using the first N POD modes to represent the entire F−δ dataset. In the case of PS–LDPE, the first three POD modes are chosen to represent the entire force–penetration evolution, which occupies the majority of the “energy” (L2-norm of the reconstructed signal after truncation compared to that of the raw data) contribution (up to 99%). Let us stress that the term “energy” here refers to a way to assess the fidelity of the captured signal after modal truncation, and not to a mechanical energy.
***U***^(1)^: the first POD mode contains the primary information on the phases. As shown in Figure 3d, the nature of each phase is remarkably well captured (PS in grey and LDPE in dark red).
***U***^(2)^: the second POD mode reveals the next-order information (e.g., phase interface). As illustrated in Figure 3e, the light grey areas at the PS–LPDE interfaces indicate that the mechanical properties at these pixels are somewhat different compared to either the PS matrix or the LDPE nanodisk.
***U***^(3)^: the third POD mode reveals even more subtle information that does not affect the first two POD modes. For instance, (as highlighted in Figure 3f) the $U^{\left(3\right)}$ map reveals regions with steep slopes, such as a scratch at the south-east side, which correlates well with similar defects revealed in SEM images. Moreover, $U^{\left(3\right)}$ also reveals the contact points of the AFM tip with the LDPE pods (Figure 3f), where the difference in the effective contact surface can be related to the observed behaviour at these pixels.
*ρ*: the effect of higher-order POD modes, n>N, has a negligible power. In this regard, the residual field, ρ(x) can be computed to locally evaluate the accuracy of POD reconstruction by using the first N POD modes. In the presented case, if only the two first POD modes are used to reconstruct the force–penetration curve for the entire ROI, the residual field $\rho_{2}\left(x\right)$ indicates a large reconstruction error up to 50%, particularly located in the region of LDPE nanopods. However, when using the first three POD modes, the reconstruction residual is significantly decreased to 8% on average, and no particular dependence is observed between the topography and residual field. A closer inspection shows that at the PS and LDPE interface, residuals remain high (about 5%), not higher than other points in the analysed region, but never small, so that the interface can still be perceived. This may result from the natural variability that is expected along the boundary of the pods both because of the different materials and the change in topography.

Instead of comparing each individual POD mode, the subspace generated by the POD modes provides a straightforward visual inspection, allowing for a more robust quantitative analysis if required. Several visualization methods can be considered.

- Using the distribution of data in the subspace $\left[U^{\left(1\right)};U^{\left(2\right)};U^{\left(3\right)}\right]$, Figure 3h shows that all pixels are grouped in different clusters.
- Alternatively, using the first two modes, $\left[U^{\left(1\right)};U^{\left(2\right)}\right]$, a 2D histogram can be plotted [22], which shows the frequency of pixel distributions having a given value of $U^{\left(1\right)}$ and $U^{\left(2\right)}$.

In these visualization methods, two main phases characterized by their mean responses (centroids) and deviations are very clearly illustrated, making the segmentation task quite simple. Furthermore, uncovering the underlying state variables that govern data distribution is equally important for modelling purposes. To fulfil this task, several manifold learning tools are presented in the following, together with their segmentation results and mechanical interpretation.

For the case of PS–LDPE, the data manifold is first considered to be composed of two major species (zero-dimensional manifold). Therefore, the clustering analysis (e.g., K-means) based on similarity measurements offers a very easy way to single out their common patterns in a collection of force–displacement responses. Furthermore, to gain further insight from higher-order parameters, the data-support manifold can be described by considering it as a curve (a curve links two clusters through a progressive transition), where either the probability model-based clustering (e.g., Gaussian mixture model) or manifold learning techniques (e.g., ISOMAP) are more adapted to achieve a detailed description.

#### 4.1.3. K-Means Clustering

**Figure 4:**
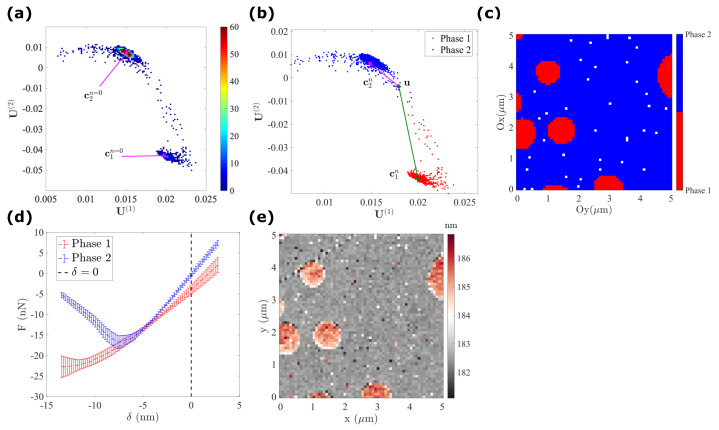
K-means clustering to segment the PS–LDPE sample into two clusters: (**a**) Initialization of two centroids with the help of a 2D histogram; (**b**) segmentation of all pixels $x^{*}$ by evaluating the distance to each centroid; (**c**) cartography of pixels via K-means clustering, where phase 1 (in red) corresponds to the LDPE nanopods and phase 2 (in blue) to the PS film; (**d**) average response of each cluster, phase 1 (LDPE) plotted in red illustrates a softer modulus and higher adhesion, phase 2 (PS) in blue shows a higher stiffness and lower adhesion; (**e**) topography map of the PS–LDPE sample.

As illustrated in Figure 4, in the subspace generated by the POD, all data points u, are mostly grouped into $N_{c}=2$ major clusters. To identify those clusters, the K-means algorithm is used. It comprises computing the centroid of each cluster $c_{k}$, where k is the cluster label, such that the total inter-cluster square distance J(c) is minimized

(9) $$J\left(c\right)=\sum_{k}\sum_{u\in C_{k}}{∥u-c_{k}∥}^{2}.$$

It is trivial to provide an initial guess of centroids for each group by visual selection of Figure 3h. Therefore, all data points are grouped into clusters according to the closest centroid (as illustrated in Figure 4b), i.e.,

(10) $$u\in C_{k}\Leftrightarrow k=\arg\min_{j=1}^{N_{c}}∥u-c_{k}∥.$$

At each iteration n, the centroid of each cluster is updated as the centre of the set of points that belongs to that cluster

(11) $$c_{k}^{n}={\langle u\rangle }_{u\in C_{k}}.$$

The process is repeated until the cost function reaches a stationary value

(12) $$\left|\frac{J\left(c^{n}\right)-J\left(c^{n-1}\right)}{J(c^{n})}\right|\leq 1\times 10^{-4}.$$

To give a proper interpretation of the clustering results, the cartography of segmentation via K-means clustering is first shown in Figure 4c. It is clear that the first phase (plotted in red) corresponds to the nanopods of the LDPE, while the second phase can be attributed to the PS film, in agreement with the topography shown in Figure 4e. Moreover, the position of each centroid in the POD subspace can be used to estimate the average behaviour of each cluster (phase), written in the form

(13) $${\tilde{F}}_{k}\left(\delta \right)=\sum_{j=1}^{N}\lambda^{\left(j\right)}c_{k}^{\left(j\right)}W^{\left(j\right)}\left(\delta \right).$$

In this expression, $c_{k}=\left(c_{k}^{\left(1\right)},c_{k}^{\left(2\right)},c_{k}^{\left(3\right)}\right)$ represents the final position of centroids for the k-th cluster in the POD subspace. The average force–indentation response of these two clusters is plotted in Figure 4d, the LDPE cluster has a softer modulus but a higher adhesion compared to the PS cluster, consistent with the already well-known mechanical properties of these two materials.

#### 4.1.4. Gaussian Mixture Model

The Gaussian Mixture Model (GMM) is another clustering algorithm that exploits an additional assumption, namely, that the data points result from the superimposition of a discrete set of components, each of which follow a Gaussian distribution.

##### Number of Components

**Figure 5:**
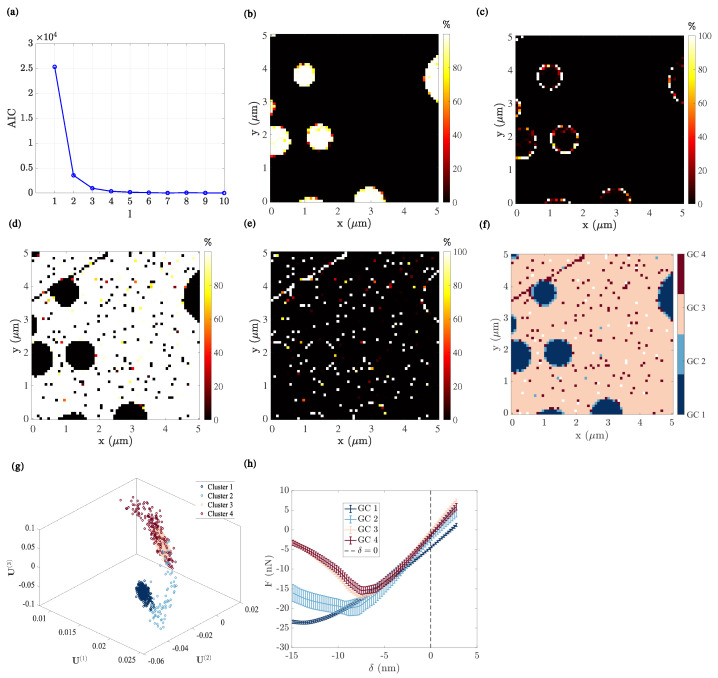
Clustering analysis applied to the u subset from the force–indentation response of the PS-LDPE sample using GMM fitting: (**a**) Akaike information content as functions of the number of Gaussian mixture components; (**b**–**e**) Map of posterior probability for the local u to result from the l-th Gaussian component; (**f**) Cartography of each GC; (**g**) Segmentation of u in the modal amplitude space; (**h**) the corresponding force–indentation curve of each GC.

The first step of the GMM analysis comprises estimating the number of Gaussian components (GCs). The optimal number of GCs may be obtained using different strategies. In the following, it is proposed to evaluate the quality of the clustering analysis through the Akaike information content (AIC) defined as

(14) $$\mathrm{AIC}\left(k\right)=2k-2\ln\hat{p}$$

where k is the number of estimated parameters in the GMM and $\hat{p}$ represents the maximum value of the likelihood function of the GMM. The elbow method comprises evaluating the AIC as a function of the total number of GMM clusters. The AIC is plotted vs. the number k of GCs in Figure 5a. When k<4, adding one additional k+1-th GC brings significant improvement in the clustering precision (lower AIC). However, for k≥4, the benefit of further model enrichment becomes negligible. Hence, k=4 is the so-called ‘elbow point’, and leads to the conclusion that, for the case of PS–LDPE, four GCs is the optimal choice.

##### GMM Clustering Analysis

The expectation maximization (EM) algorithm is deployed to determine the parameters $\theta_{k}$ of the probability density distribution function (PDF) for each GC, k. From the latter, the posterior probability for any given u from the l-th GC, p(k=l|u), for l=1, 2, 3 and 4, can be estimated via the Bayes theorem.

Finally, for all pixels $x^{★}$ inside the ROI, each pixel can be assigned to a particular GC, namely, one which maximizes the posterior probability, i.e.,

(15) $$x\in G_{l}\Leftrightarrow l=\arg\max_{k=1}^{4}p\left(k|u(x)\right)\;.$$

Here, p(k|u) represents the posterior probability for a pixel x characterized by the modal amplitudes u(x) belonging to the k-th GC.

In Figure 5b–e, the maps of posterior probability for each GC are plotted. To facilitate the mechanical interpretation of the Gaussian mixture clustering results, the cartography of GCs, the assignment in the POD subspace, and the mean force–indentation response of each cluster are plotted in Figure 5f–h, respectively. Several comments and mechanical interpretations can be drawn in this context:

(1) The first GC (Figure 5b) corresponds to the LDPE nanodisks, with a posterior probability very close to 1. Consistently, it exhibits the lowest force–indentation slope (highest compliance) among all clusters. Upon unloading, the position δ for which F=0 is the highest indicates a marked viscous behaviour (Figure 5h).
(2) The second GC (Figure 5c) only shows a large posterior probability at the PS and LDPE interface. Correspondingly, its response is characterized by a higher stiffness, lower adhesion, and lower viscosity together with a much higher scatter of values (Figure 5h).
(3) The third GC (Figure 5d) can be interpreted as consisting of the PS film. Consistently, it shows the highest force–indentation slope, almost no viscosity, and very low adhesion (see (Figure 5h)). Moreover, its mechanical response is very precisely defined, with a very low scatter.
(4) The fourth GC (Figure 5e) is more difficult to interpret. Indeed, it shows a mechanical response very similar to that of the third GC, and indeed in the u space, these two components exhibit a wide overlap. The main difference comes from the much wider scatter of the fourth GC. This may be the result of the fact that the distribution of modal amplitudes for the PS film is not Gaussian. However, in the region where this component does not overlap with the third one, (well captured in the GC labelling), it highlights the topography contribution inside the PS film, revealing the presence of a surface defect (in particular, a scratch in the north-west corner). The underlying topography variation results in a larger scatter in terms of force indentation. It can then be concluded that sharp topography variations of the PS film, such as scratches, can be distinguished from the plain flat surface, mostly because of their scatter, and this subtle property is well captured by the GMM procedure.

The mechanical interpretation for the clustering is consistent with the a priori knowledge of the features and phases of the tested sample (i.e., the difference in mechanical properties between PS and LDPE). Moreover, not only the present analysis highlights the principal state variables from the tested sample (i.e., the nature of the PS and LPDE phases), but it also reveals the contribution of higher-order parameters (such as the interface between phases, and topographical defects).

#### 4.1.5. Manifold Reparametrization

With the visual inspection of the POD subspace $u=\{U^{\left(1\right)},U^{\left(2\right)},U^{\left(3\right)}\}$, the data points form a narrow but non-flat surface. The embedding dimensionality, three, is somewhat misleading since the data are supported by a topologically two-dimensional manifold; hence, only two state variables are sufficient to describe the wealth of observed mechanical responses.

Herein, the isometric feature mapping (ISOMAP) tool is chosen to compute the two state variables θ(x),ρ(x)

(16) $$\begin{array}{c} \theta (x),\rho (x)=\mathrm{ISOMAP}(u,N=2). \end{array}$$

**Figure 6:**
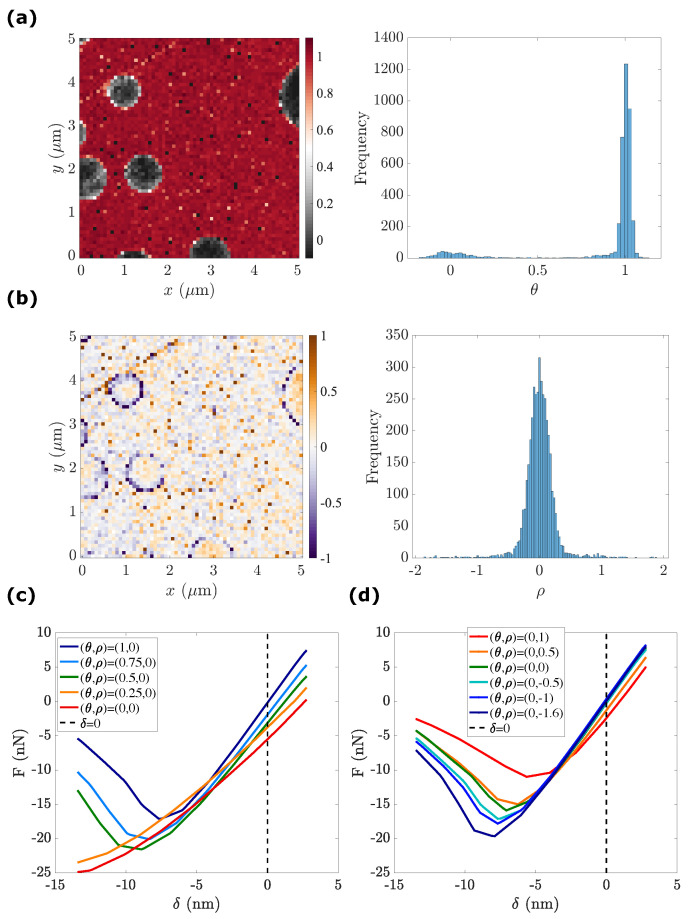
Manifold re-parametrization: (**a**,**b**) the state variables map, (θ,ρ), for PS–LDPE and their associated histogram. (**c**,**d**) The reconstructed force–indentation response using state variables: (**c**) influence of θ; (**d**) influence of ρ.

After the manifold re-parametrization, the first state variable θ exhibits a bimodal distribution (Figure 6a). In order to give a mechanical interpretation of θ, a re-interpolation of the force–indentation curves with several representative θ values is plotted in Figure 6c. It is to be highlighted that θ remarkably captures the nature of each phase:

- θ=0 corresponds to the LPDE phase (nanodisk deposit) with the lowest force–indentation slope and highest adhesion.
- θ=1 corresponds to the PS phase (film matrix), with the highest stiffness and lowest adhesion.
- 0.25≤θ≤0.75 corresponds to the PS–LDPE interfaces, with an intermediate value of both stiffness and adhesion. As expected, with an increasing value of θ, we observe an increase in stiffness and a decrease in adhesion.

In parallel, the secondary state variable ρ shows a single peak distribution (Figure 6b). Moreover, most pixels with a large non-zero value of ρ are located inside the PS film matrix (θ=1). Therefore, the mechanical interpretation of the second state variable ρ is carried out by setting the first state variable to θ=1. As shown in Figure 6b, when compared to the standard PS response where the film is smooth (θ,ρ)=(1,0), a surface scratch in PS film can be characterized by (θ,ρ)=(1,1), and it exhibits a similar stiffness but a lower adhesion. This is consistent with the fact that topographical features lead to a decrease in adhesion.

In turn, the PS–LDPE interface, which can be described by (θ,ρ)=(1,−1), exhibits a slight increase in adhesive force. This increase in adhesion can be explained by the fact that the size of the AFM tip is non-negligible (l≃10nm); therefore, the adhesion measured at a given pixel is actually a 2D surface integral of the local adhesion force.

Several additional remarks are in order:

- For any given pixel x, when ρ is around zero (|ρ|≤0.25), the surface can be considered as smooth and when ρ is shifting away from the mid-width of the Gaussian distribution (|ρ|≥0.25), the surface becomes rough (with significant topographical variations).
- Interestingly, different types of topographical features are associated with different values of ρ, i.e.,
  – surface defects (scratch): ρ≃1;
  – phase interface (frontal interface between the LDPE nanodisk and AFM tip): −2≤ρ≤−0.5.

In this regard, the second state variable captures the influence of topographical features irrespective of the underlying mechanical response. Thus, it can be also inferred that topographical features impact the force–penetration response in a different manner depending on whether they lie at the material’s interface or at surface defects. These subtle differences can be related to either the difference in the effective contact area or the viscoelasticity at the tip–sample interface.

### 4.2. Carbon–Iron Particles with a PDMS Binder

The second analysed sample is a magnetorheological elastomer (MRE), or simply, a composite material consisting of a polymer matrix (PDMS) and mechanically stiff, carbonyl–iron particles (CIP) with a mean radius of about ∼3 μm. Details on its manufacturing process may be found in [36]. The proposed analysis may prove extremely useful to understand the surface properties of such MRE materials since recently they have been used in the form of films [37] and membranes [38], and were shown to exhibit significant non-linear viscoelastic properties [39,40].

**Figure 7:**
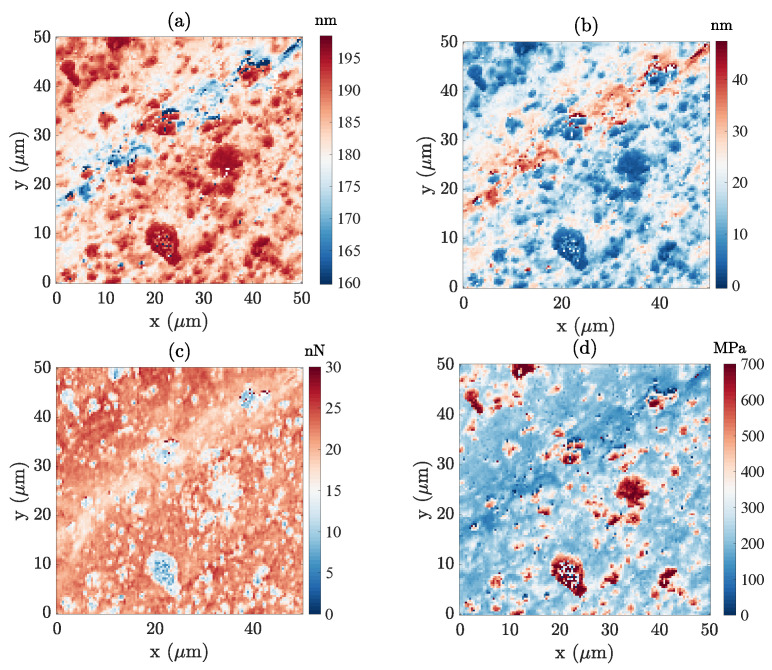
(**a**) Topography using the proposed definition of elevation at zero force after the first contact; (**b**–**d**) three built-in Bruker’s PF-QNM results: (**b**) Maximum indentation; (**c**) adhesion; and (**d**) apparent elastic modulus (based on the Sneddon model).

**Figure 8:**
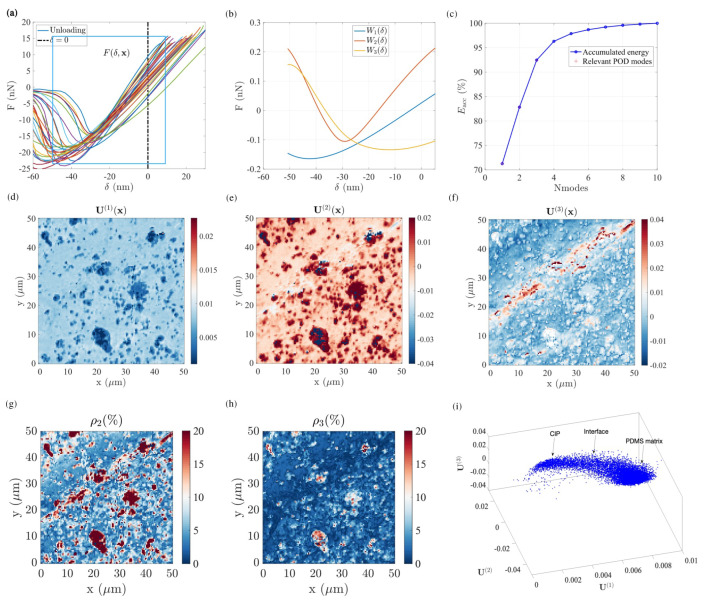
(**a**) Arbitrarily selected force–indentation response at various pixels during retraction: The rectangle indicates the region selected for POD analysis; (**b**) the first three POD elementary force modes as a function of the penetrations; (**c**) accumulated “energy” (L2-norm of reconstructed signal) as a function of the number of POD modes, where the first three POD modes represent 94% of the total energy; (**d**) the first POD spatial mode reveals the nature of the phase at each pixel; the second (**e**) and third (**f**) POD modes emphasize the more subtle pieces of information, such as the PS–LPDE interfaces and shows higher-order features related to surface roughness; (**g**,**h**) residual error resulting from only keeping the first N modes to describe the force–indentation response at each pixel, N=1 and 3, respectively. (**i**) Three-dimensional point distribution in the POD modal amplitude space $\left[U^{\left(1\right)};U^{\left(2\right)};U^{\left(3\right)}\right]$.

A surface of $50\;\mu m^{2}$ is scanned with 128×128 PF-QNM measurement points, at 2 kHz. From the measured signal, using the above-mentioned definition of topography as the elevation for which the contact force vanishes, a topography map can be computed, as shown in Figure 7a. The built-in PF-QNM module provides different characterizations, as displayed in Figure 7b–d. These results allow for a comparison with the results obtained from the POD analysis (Figure 8a–i). The surface displays a major scratch, and salient asperities that can be identified as CIP from SEM images, where the large atomic number of CIPs gives rise to very bright spots (as shown in the appendix of [22]).

Figure 8a clearly shows that the mechanical response gradually evolves, with obvious signs of viscosity and adhesion in contrast with the previous sample. This complexity makes segmentation, and a fortiori identification of the underlying phases, a very arduous task. In particular, as highlighted in the topography map in Figure 7a, a major groove (along the first diagonal) is present in the analysed region. This topography, chiefly its sharp gradient, induces indentations of varying severity and, in turn, may affect the extraction of the elastic modulus and adhesion mapping.

Following the same POD procedure presented in the previous section, a window is selected in the unloading Regime III (Figure 8a) with δ ranging from ∼−50 nm to ∼5 nm. In this initial dataset, after the POD analysis, the first three modes are retained. Their mechanical responses are shown in Figure 8b. Their contribution amounts to $\tau_{1}=0.73>\tau_{2}=0.11>\tau_{3}=0.09$, describing approximately 94% of the energy (L2-norm) of the original F−δ data, as shown in Figure 8c.

Let us emphasize that the first mode $U^{\left(1\right)}$ (Figure 8d) already allows for a clear distinction between the PDMS matrix (light blue) and the CIP inclusions (darker spots), where manifested topography is not present at all. In particular, aggregates of several particles exceeding 10 μm (i.e., 3–4 times the particle size) are clearly visible. Figure 8d illustrates the difference in sensitivity of the AFM–POD analysis compared with the SEM imaging, the former probe depths of the order of particles or clusters of particles, while the latter reveals microstructures at the scale of the electronic penetration depth.

The second mode (Figure 8e) in the present case does not substantially exhibit different features when compared to the first one. In fact, one may note that the CIPs now have a much larger weight than the soft matrix, i.e., in opposition to the first mode. One of the most striking features of these first two modes is that they appear to not be influenced by topography, and hence the identity of the phases is very easily observed. This is in contrast with all the different outputs of the classical analysis (as shown in Figure 7a–d), where the surface topography is very clearly mingled with the phase contrast.

The residual amplitude after removing the contributions of the first two modes is shown in Figure 8g. Clearly, features from the surface, such as the topography, show that the two modes are insufficient (even if 83% of the energy L2-norm of the signal is gathered).

The third mode (Figure 8f) reveals a more subtle variation in the mechanical response, which now is strongly correlated with topography. In particular, the deep groove from the bottom-left to top-right is clearly emphasized. In the literature, the impact of topography on the apparent adhesion has been well studied. Sharp slope changes (high curvatures) modify the probed adhesion [41]. This is consistent with the above results as well as those processed by the built-in Bruker AFM software; however, the POD analysis shows that this effect can clearly be distinguished from other features, such as the nature of the phase at the surface. The corresponding residuals after the three modes are displayed in Figure 8h. Some information is still present but the large-scale features, such as the large groove, has been erased.

When considering the distribution of the different modal amplitudes in the 3D space $\left[U^{\left(1\right)};U^{\left(2\right)};U^{\left(3\right)}\right]$, it is interesting to note that the data are not clustered in discrete clusters, but rather they show a gradual evolution (Figure 8i).

**Figure 9:**
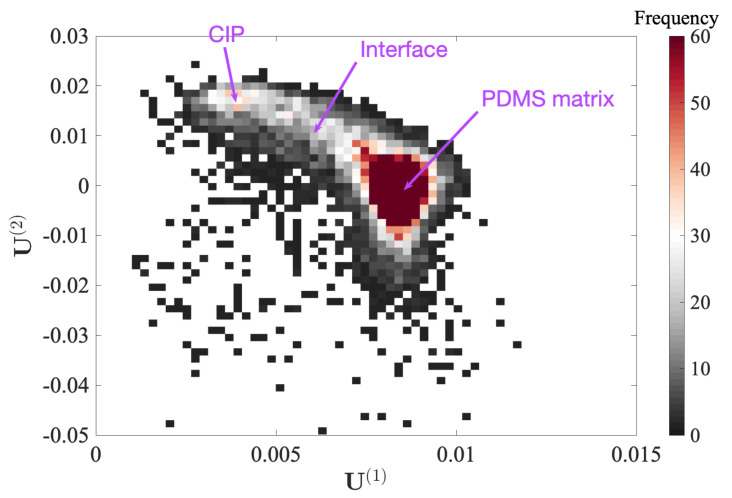
Two-dimensional histogram of the amplitudes in the POD subspace $\left[U^{\left(1\right)},U^{\left(2\right)}\right]$.

Because the topography has mostly been erased from the first two POD modes, the subspace $\left[U^{\left(1\right)};U^{\left(2\right)}\right]$, (Figure 9), appears to be a natural space for phase segmentation.

In the cloud of points given by the two modal amplitudes for each “pixel”, the two extreme points are more densely populated, and correspond to the PDMS and CIP phases, as discussed earlier from the first mode amplitude. However, the gradual transition between these two spots shows that the mechanical properties vary continuously. A natural interpretation is that this transition is due to particles being buried within the PDMS matrix at different (shallow) depths. This is a clear demonstration that the POD analysis is a powerful tool to sort the hierarchy of different features which contribute to the overall mechanical response (nature of the phase, topography, …) and that it is very efficient in describing each pure phase or their combination.

For the PDMS–CIP, due to the progressive transition between the two phases, it becomes less relevant to resort to K-means clustering. Therefore, only GMM is used as a clustering analysis.

#### 4.2.1. Gaussian Mixture Model Fitting

**Figure 10:**
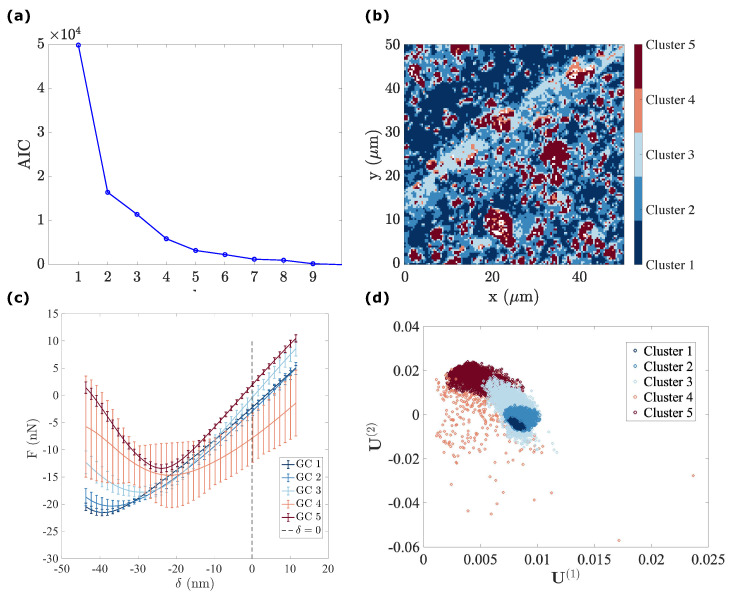
Clustering results applied to the u subset from the PDMS–CIP sample: (**a**) AIC against the number of used GCs, the optimal number of GC to be adopted is $l^{\mathrm{opt}}=5$; (**b**) cartography of each GC; (**c**) mean force–penetration response for each GC; (**d**) cluster segmentation of u.

The same GMM clustering analysis procedure presented in Section 4.1.4 is applied here. In Figure 10a, after plotting the AIC vs. the number of used GCs, a GMM fitting with five components is selected. After performing the EM algorithm, the parameters for each GC are identified ($\theta_{l}$, l=1,2,⋯,5), allowing for the calculation of the PDF for each cluster. Finally, using the Bayes theorem, the probability for any given u arising from the l-th GC can be estimated.

The segmentation results are illustrated in Figure 10b–d. Combined with the representative force–indentation curve belonging to each GC (see Figure 10c), several conclusions can be drawn:

- GC 1, 2 and 3 (PDMS matrix): the first three components plotted in dark, medium and light blue represent the majority of the PDMS matrix with a very comparable soft modulus and small viscosity. Their adhesion tends to decrease from GC 1 to GC 3. Moreover, the spatial distribution of the components suggests that the main difference between these three components is due to topography, from a plateau level for GC 1, down to deep valleys (scratches) for GC 3. This observation may be due to the maximum penetration being high for GC 1 and low for GC 3, and the effectively mobilized adhesion may be due to the indentation depth.
- GC 4 (PDMS–CIP interface): as illustrated in Figure 10d, most of the data points that belong to the forth cluster are very widely scattered, and hence they may be difficult to interpret. However, their location is clearly in very close vicinity to the CIP or CIP aggregates. The most salient feature of their mechanical response (apart from the expected broad dispersion) is their high viscosity. This may result from the enhanced strain and strain rates in the PDMS phase when it is confined within a CIP cluster, resulting in an amplification of the viscous contribution.
- GC 5 (CIP): The fifth component (plotted in dark red) exhibits the highest force–indentation slope, low adhesion, and low viscosity. This observation is consistent with their morphology, supporting the fact that it corresponds to CIPs at the surface.

It should be noted that the different Gaussians identified for the GMM model have significant overlap. Therefore, as one can easily measure the probability of belonging to one phase or another, it is easy to see that for many points (pixels), the labelling is ambiguous. This implies that segmentation is somehow artificial, and hence a different, more progressive reading of the data is more relevant. Physically, this may result from, e.g., a layer of PDMS on top of the CIPs. Depending on the polymer thickness, the response may continuously change from that of CIPs (no coating) to that of the matrix (thick coating). The following section proposes such an analysis.

#### 4.2.2. Manifold Re-Parametrization

In the case of PDMS–CIP, the manifold can be read as one main backbone curve or two transversal variations (leading to three parameters). Hence, ISOMAP feature analysis is carried out in three-dimensions introduced as state variables

(17) θ(x),ρ(x),ψ(x)=ISOMAP(u,N=3).

**Figure 11:**
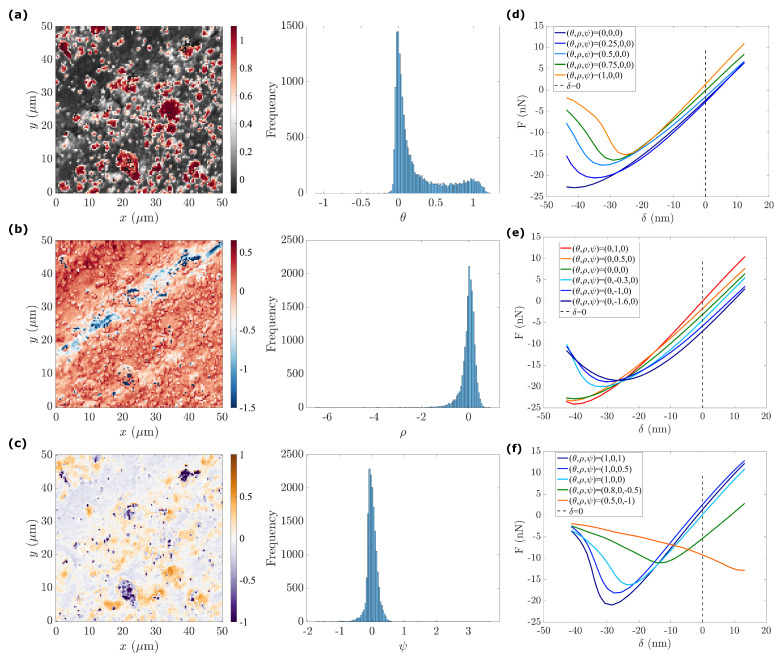
Manifold re-parametrization applied to the u subset for the PDMS–CIP specimen: (**a**–**c**) Maps of the three state variables, (θ,ρ,ψ) and their associated histogram; (**d**–**f**) the reconstructed force–indentation responses using different state variables (θ,ρ,ψ) for the PDMS–CIP specimen.

The maps of state variables and their associated histograms are illustrated in Figure 11. The main state variable is θ, exhibiting a bimodal distribution. The two other state variables, ρ and ψ, show a single peak distribution. Nevertheless, a re-interpolation of the force–indentation response using these state variables (Figure 11d–f) is required to provide a proper mechanical interpretation of the three presented maps. We discuss each of these parameters in the following:

- θ: We study the effect θ by setting (ρ,ψ) to their mean value (0,0), respectively. By varying the first state variable (θ=0,0.25,0.5,0.75,1), we observe a progressive increase in the stiffness (F−δ slope) and a decrease in adhesion (see Figure 11e). The response of θ=1 exhibits the highest force–indentation slope, a near-zero dissipation, and the lowest adhesion, corresponding to the presence of CIPs. The response of θ=0 shows the lowest apparent modulus and highest adhesion, corresponding to the presence of a viscous and adhesive PDMS matrix. As for all intermediate values, θ=0.25,0.5,0.75, the corresponding response indicates a progressive evolution, especially in adhesion, implying a decrease in the influence of the PDMS matrix as θ increases.
- ρ: The second state variable map, ρ, (Figure 11b) correlates well with surface topography (Figure 8c). From deep in the valley where ρ∼−1.6, the mean surface ρ∼0, and to the most salient peaks where ρ∼1, the surface topography becomes the main characteristic captured by ρ. Force indentation responses with different values of ρ (see Figure 11e) were compared by setting the two other state variables to 0 (θ=0,ψ=0) (focusing mainly on the PDMS matrix). Compared to the standard PDMS matrix surface, (θ,ρ,ψ)=(0,0,0), the force–indentation response in the valley, where (θ,ρ,ψ)=(0,−0.5,0), leads to a significant decrease in the apparent adhesion while maintaining a similar stiffness. This observation is consistent with previous observations of the GMM analysis, where it was argued that for deep valleys, the penetration distance and effective contact radius is somewhat smaller than for higher regions, and hence the apparent adhesion is expected to be reduced.
  Furthermore, focusing on the deepest positions inside the valley (ρ varying from −1.6 to −1), an increasing viscosity is observed. This increasing viscosity can be interpreted as an artefact due to the tip velocity. Note that for a local scan inside a deep valley where the topography is much lower than the average surface (see Figure 8d), the tip should be subjected to an increased velocity to comply with the required AFM scanning frequency at f=2 kHz. To further support this interpretation, Figure 8d shows that the largest penetration distances (excluded from the analysis because of our selected range of δ) are associated with the largest apparent viscosity values. On the contrary, when ρ is positive (ρ=0.5,1) the force–indentation response shows a similar adhesion to a plain PDMS surface ρ=0 with increased stiffness, implying the presence of CIPs buried just below the PDMS surface. Therefore, the rho map reveals the impact of surface topography on the mechanical response (and in some respect also on the acquisition artefacts).
- ψ: The distribution of ψ follows a single peak distribution. For this state variable, values very different from 0 are mostly concentrated in large CIPs or CIP aggregates. Thus, in setting θ=1, the main effect of increasing ψ from zero is a significant variation in adhesion. This may be interpreted as the effect of either particle immersion or being covered by a thin layer of PDMS that changes the adhesion by a large amount without altering stiffness (over the penetration range).
  Large negative ψ≤−0.5 values (purple-blue in Figure 11c) correspond with pixels located either very deep in the valley or very high at salient peaks. They also coincide with regions where ρ≤−1.5. When compared to the previous analyses, all these pixels correspond to the fourth GC, and in terms of modes, they could be identified in modes 1 and 2 as data points that do not exhibit smooth continuity with their surrounding. When the force–displacement response is computed for two sets of values (θ,ρ,ψ)=(0.8,0,−0.5) and (0.5,0,−1), as shown in Figure 11f), odd non-physical shapes are obtained. All these observations support the fact that these measurements were unreliable, as previously observed, and when forced to organize these data in a sensible way, very unusual values and increased scattering of the various properties may be obtained. In this view, the obtained range of values for ψ mainly show pixels that should be discarded rather than providing a trustworthy measurement of a given mechanical quantity.

## 5. Conclusions

The recently popularized PF-AFM mode greatly improves the scanning frequency of AFM mapping and thus permits the recording of large datasets containing many features in a short acquisition time. Though harvesting such an abundance of data is highly beneficial to locally characterize the observed specimen, handling the measurement of well-defined mechanical properties individually at each pixel location becomes a progressively more challenging problem. Furthermore, the validity of the modelling used to fit the data may be questioned, especially when assessing viscoelastic and non-linear polymer materials.

To circumvent these difficulties, the proposed combined model reduction and manifold learning algorithm allows for identifying generalized common patterns and interpretation of the mechanical properties in both samples analysed in this study.

First, the efficiency of the POD model reduction technique in achieving a low-dimensional representation of the initial data has been demonstrated. The model reduction process can seamlessly separate the nature of phases from other higher-order parameters (topography or viscosity) in the POD subspace, allowing the analyst to visually inspect the data manifold, which is of great practical interest for robust qualitative analysis.

Second, the underlying state variables can be fully uncovered through clustering analysis or manifold learning. Proper adapted manifold learning tools can be pre-determined accordingly based on the dimensionality of the observed data (modal amplitudes) during the previous “visual inspection” step.

Specifically, the proposed manifold learning algorithm is a conceptual approach to hyper-reduction applied to PF-QNM AFM data. In fact, the reconstructed force–indentation curves with various state variables may be seen as a further step towards generating representative data, thereby bridging the gap with numerical simulations, where these state variables can equally be used. The optimality of manifold learning (the number of state variables used to represent the data) is a major advantage of the proposed approach.

Furthermore, it is important to underline the agnostic nature of the proposed algorithms since they do not rely on a specific model but may very well be approximate. This is both a “strength” and a “weakness”. A strength because inappropriate modelling can be avoided, and a weakness because the reduced response still has to be interpreted in terms of the intrinsic mechanical properties. However, the reduced and parameterized representation of the response is much less intensive, and hence allows for much more sophisticated and realistic modelling to capture the quantitative physical meaning of the characterization.

## Data Availability

Data are available from the authors upon request.
